# Integrated Health and Social Home Care Services in Catalonia: Professionals’ Perception of its Implementation, Barriers, and Facilitators

**DOI:** 10.5334/ijic.7530

**Published:** 2024-04-26

**Authors:** Pilar Hilarión, Anna Vila, Joan C. Contel, Sebastià J. Santaeugènia, Jordi Amblàs-Novellas, Rosa Suñol, Conxita Barbeta, Aina Plaza, Emili Vela

**Affiliations:** 1 Avedis Donabedian Research Institute (FAD), Spain; 2 Universitat Autònoma de Barcelona, Barcelona, Spain; 3 Health Services Research Network on Chronic Diseases (REDISSEC), Spain; 4 Network for Research on Chronicity, Primary Care, and Health Promotion (RICAPPS), Spain; 5 Integrated Social and Health Care Program, Department of Health and Department of Social Rights, Generalitat de Catalunya, Barcelona, Spain; 6 General Directorate of Personal Autonomy and Disability, Department of Social Rights, Generalitat de Catalunya, Barcelona, Spain; 7 General Directorate of Health Planning, Department of Health, Generalitat de Catalunya, Barcelona, Spain; 8 Central Catalonia Chronicity Research Group (C3RG), Centre for Health and Social Care Research (CESS), University of Vic—Central University of Catalonia (UVIC-UCC), Barcelona, Spain; 9 Intermediate Care Director, Parc Sanitari Sant Joan de Déu, Sant Boi de Llobregat, Spain; 10 Àrea de Sistemes d’Informació, Servei Català de la Salut, Barcelona, Spain; 11 Digitalization for the Sustainability of the Healthcare System (DS3), IDIBELL, Barcelona, Spain

**Keywords:** home care services, home care, integrated care, health care, social care, barriers and facilitators

## Abstract

**Introduction:**

This study aimed to assess the implementation of integrated social and health home care services (HCS) offered by the Government of Catalonia, and to identify the main barriers and facilitators of integrated HCS.

**Methods:**

Analysis of the degree of implementation of integrated social and health HCS perceived by social care services (SCS) and primary health care centers (PHCs) between December 2020 and June 2021 in two phases. First, the perception of integration by social workers within SCS and PHCs was assessed using a screening questionnaire. Then, SCS in counties with the highest integration scores received a customized questionnaire for an in-depth assessment.

**Results:**

A total of 105 (100%) SCS and 94 (25%) PHCs answered the screening questionnaire, and 48 (45.7%) SCS received a customized questionnaire. The most frequent barrier identified was the lack of shared protocols, with the most frequent facilitator being the recognition of the importance of integrated HCS.

**Conclusions:**

Our study showed that the degree of implementation of integrated health and social HCS offered by the Government of Catalonia was perceived as low. The identified barriers and facilitators can be used to facilitate such implementation. Further studies should include professionals other than social workers in PHC assessments.

## Introduction

Most countries in the world are experiencing an improvement of survival beyond the age of 65, along with an increase in the number of adults aged 65 years or older, a trend that is expected to continue [1]. Consequently, in OECD countries, the population aged 65 and older is expected to account for 26.7% of the whole population by 2050 [2,3]. In the case of Catalonia (North-east of Spain), which has one of the most intensively aging populations globally, this age group is projected to increase to 30.5% by 2050 [4]. This demographic shift is expected to increase the demand for long-term care in the next decades, including services to support home-based care for older adults [5], so home care services (HCS) policies could become a priority in the next years. Moreover, HCS are an important resource to promote the autonomy of individuals of all ages [6].

The main outcomes of home care include promoting the care recipients’ quality of life and functional independence in the domains of social, personal care, and mobility, but also minimizing the utilization of other services, such as hospital care, admission in other settings (e.g., nursing homes), and acute emergency admissions [7,8]. For this purpose, current home care models include health and social services in the home environment [8] delivered by a wide range of professionals (home care assistants, nurses, social workers, physical therapists, occupational therapists, physicians, etc.) along with informal caregivers (e.g., relatives or volunteers) [8,9]. In most European countries, formal home care is provided by the social and health care systems. Usually, the social system provides home help services such as household duties, personal care, and other activities (e.g., socializing or going for walks), whereas the health care system mainly provides nursing care, occupational therapy, and physiotherapy [8].

The delivery of both social and healthcare services from different sectors must be coordinated and complementary to improve accessibility for care recipients, achieve better outcomes in terms of user experience, and avoid a waste of human and financial resources, such as that resulting from duplication of services provided by different organizations [10,11]. Integration, which is defined as “a coherent set of methods and models on the funding, administrative, organizational, service delivery, and clinical levels designed to create connectivity, alignment, and collaboration within and between the cure and care sectors” [12], is an important aspect to consider in Home Care Services (HCS), given that it enhances the quality of care, quality of life, and satisfaction of users, and improves the efficiency of the system [12].

In Catalonia, the Department of Social Rights [13] and the Department of Health [14] provide social and healthcare services to cover the needs of its population (about 7.7 million inhabitants [15]). Both Departments have been working together since 2014 to create a single strategy of integrated social and health HCS with the aim to benefit care recipients –improving their health, quality of life, and care experience, and preventing or postponing dependency– and guarantee proper use of social and healthcare services. Catalan social services in the home setting mainly provide help in personal care, but also deliver support in household tasks and teleassistance [16]. They are funded both by the Department of Social Rights and the local governments [17], and are mostly delivered by outsourced companies. In addition, health services in the home setting include disease prevention and health promotion activities involving primary health professionals such as physicians, nurses, and social workers [18]; hospital at home, in which hospital health care professionals deliver an active treatment to a patient who would otherwise need acute care in a hospital [19]; and end-of-life care through multidisciplinary and specialized teams [20]. In Catalonia, primary and community care is delivered through primary health centers (PHCs), whereas social care is provided by social care services (SCS). PHCs and SCS are managed by different metropolitan or county councils [21,22]; counties are administrative geographic areas comprising one SCS and, usually, several PHC centers. A comprehensive model for integrating social and health care and a joint evaluation framework were developed with over 70 representatives of all stakeholders [23,24].

In the last years, numerous integrated care initiatives have been developed inside and outside Europe, adding important knowledge to the field. However, evidence for the effectiveness of integrated health and social care programs for people living at home remains rather inconsistent [9,25]. Therefore, this study aims to contribute to bridging this gap by assessing the perception of social care professionals about the degree of implementation of integrated social and health HCS offered by the Government of Catalonia through SCS and PHCs and to identify the main barriers and facilitators of integrated social and health HCS in Catalonia.

## Methods

### Study Design

The analysis of the degree of implementation of integrated social and health HCS offered by the Government of Catalonia was performed between December 2020 and June 2021. It was conducted in the context of the Integrated Care Program of Catalonia, which was developed according to a conceptual framework based on a snowball literature review of over 300 documents [26], an integrated care model, and an evaluation framework with the corresponding indicators and measurement elements [23,24].

**Figure 1:**
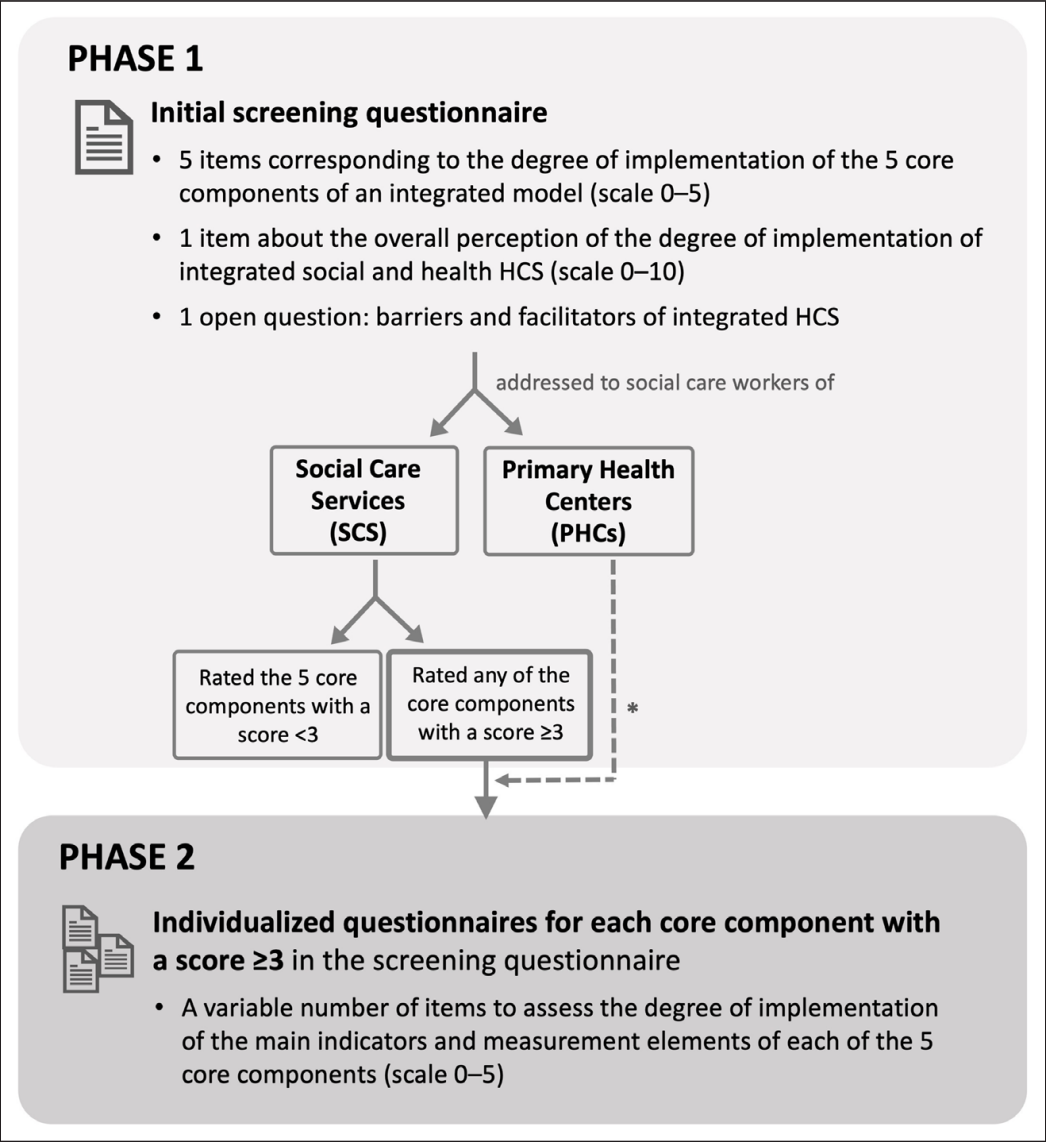
Summary of the study design and questionnaires. HCS, home care services; PHCs, primary health centers; SCS, social care services. *SCS teams were asked to answer the individualized questionnaire together with PHC teams of their council to the extent possible.

The work was performed in two phases. First, the overall perception of social workers within SCS and PHCs regarding the degree of implementation of integrated HCS was evaluated. In the second phase, those SCS with the highest scores in the first phase were selected for a more detailed assessment of the perception of the degree of implementation of integrated HCS through an in-depth questionnaire. In this second phase, SCS teams were asked to answer the questionnaire together with PHC teams of their council to the extent possible (Figure 1).

The study was conducted in accordance with the Ethical Principles for Medical Research Involving Human Subjects of the Helsinki Declaration and the local Personal Data Protection Law (LOPD 15/1999) and approved by the Research Ethics Committee of the University of Vic/Central University of Catalonia (UVIC-UCC) (reference number 176/2021).

### Data Collection and Measures

The information was obtained through two questionnaires addressed to social care professionals working in SCS and PHCs of Catalonia, who were selected as reference professionals because they play a crucial role in the coordination processes of integrated care. In the first phase, we used an initial screening questionnaire that included five items corresponding to the five core components previously established in the evaluation framework: 1) individual assessment of integrated social and health care; 2) single individual plan for integrated social and health care, built collaboratively and accessible for social and health care professionals; 3) shared protocols across health and social services; 4) coordination between social and health multidisciplinary teams; and 5) integrated portfolio services with joint social and health HCS projects. These items had to be answered using a scale from 0 (absence of integration) to 5 (excellent integration). Another item assessed, on a scale from 0 (absence of integration) to 10 (excellent integration), was the overall perception of social care teams regarding the degree of implementation of integrated social and health HCS. The screening questionnaire also included an open question regarding the main barriers and facilitators of integrated HCS in their working territory, and the identifying data of the corresponding SCS or PHC (Figure 1, Appendix 1). The screening questionnaire was sent between December 2020 and April 2021 to all SCS coordinators and all reference professionals for health social work at PHCs in Catalonia (105 and 376, respectively), who were asked to express the vision of the whole team to the extent possible. This questionnaire was designed for quick completion via e-mail, for SCS, or through an electronic form in the EUSurvey platform, in the case of PHCs.

The questionnaire used in the second phase aimed to retrieve the perceived degree of compliance of the indicators and measurement elements of the five core components, to be answered using a scale from 0 (absence) to 5 (excellent) (Appendix 2). Table 1 shows the selected indicators for each core component, and the detailed measurement elements can be seen in Tables S1–S5 (Supplementary file 1). This questionnaire was sent to reference professionals in counties that had rated any of the five items of the screening questionnaire with a score ≥3 out of 5 (Figure 1) between February and April 2021 by e-mail. Additionally, two reminders could be sent by e-mail or phone if needed. The evaluation framework and indicators description were available to guide the professionals while answering this questionnaire, along with video calls in case interpretation doubts arose.

**Table 1:** Indicators of the five core components of the model.


COMPONENT 1: “INDIVIDUAL ASSESSMENT OF INTEGRATED SOCIAL AND HEALTH CARE”	

1.	Values and preferences of the person

2.	Functional and instrumental autonomy (basic ADLs, IADLs)

3.	Need for support in decision making

4.	Multidimensional assessment of the needs of the care recipient

5.	Social and family situation and environment

6.	Evaluation of the person’s health situation

7.	Detection of risks related to the person

8.	Protective and resilience factors

9.	Screening for frailty

10.	Safe use of medication at home

11.	Safe use of equipment and other technology in the home

12.	Support and occupational therapy resources

13.	Evaluation of care providers

14.	Conditions of the home

15.	Use of resources and services in the home: remote assistance, home health workers, respiratory physiotherapy, physical/occupational therapy, home oxygen therapy, speech therapy, etc.

16.	Primary support received by the person and family or care providing environment

17.	Existence of an advanced care plan, especially in the case of ACPs

18.	Ethical implications of the care process

19.	Assessment of the person’s quality of life, conducted using a quality-of-life assessment tool or scale

20.	The initial and subsequent assessments of the person meet timing and accessibility requirements

**COMPONENT 2: “SINGLE INDIVIDUAL PLAN FOR INTEGRATED SOCIAL AND HEALTH CARE”**	

1.	The person’s care plan is unique

2.	List of needs or problems that require intervention

3.	Definition of objectives agreed upon with any care providers from other spheres

4.	Specification of the interventions and strategies that will be carried out

5.	Specification of the criteria that will be used to evaluate the results

6.	The plan is jointly prepared with the person and the team

7.	The plan is implemented from the very start of the care service, and it is reassessed within the first 6 weeks and at least once a year

8.	The person can view the plan and keeps the current and up-to-date care plan

9.	The plan includes actions by the professionals from the various disciplines and home services that visit the person at home

10.	The plan is accompanied by a home information file specifying the key agreements and aspects to be taken into account in relation to the care recipient and their family

**COMPONENT 3: “SHARED PROTOCOLS ACROSS HEALTH AND SOCIAL SERVICES”**	

1.	Definition of the systems for organizing the teams according to the territory of action

2.	Collaborative planning of the service among the agents involved

3.	Systems for allocating cases and assigning the workload

4.	Interdisciplinary and multi-agency composition of the services included in the portfolio of the home care teams

5.	Communication and messaging system for the practitioners involved in the care process

6.	Assignment of lead and co-lead caregivers for the person

7.	The team has access to the electronic case tracking system

8.	Existence of agile mechanisms for resolving any differences or conflicts of criteria arising between professionals and organizations

9.	Existence of shared protocols for HCS

10.	Shared care routes for the integrated care service

**COMPONENT 4: “COORDINATION BETWEEN SOCIAL AND HEALTH MULTIDISCIPLINARY TEAMS”**	

1.	Existence and application of a territorial functional plan that ensures the delivery of integrated care

2.	Conducting case conferences planned jointly between the social and health care teams

3.	Responses to enquiries raised between the different parties involved in the care process

4.	Information provided in the person’s transitions between different services

5.	Management of differences of opinion among the teams in accordance with established procedures

**COMPONENT 5: “INTEGRATED PORTFOLIO OF SERVICES WITH JOINT SOCIAL AND HEALTH HCS PROJECTS”**	

1.	Existence of a portfolio of social and health HCS

2.	Description of the catalog of services

3.	Periodic assessment of the programs described in the catalog of services

4.	Existence of a personal platform or folder where the person and caregiver can interact with the key professionals


ACPs, advanced chronic patients; ADLs, activities of daily living; HCS, home care services; IADLs, instrumental activities of daily living.

All the questionnaires used in this study were approved after following three main steps: 1) agreement on the items covered by the questionnaires; 2) comprehension and alpha tests of the questionnaires with professionals knowledgeable about social and health care services; and 3) pilot study in three teams of SCS to assess comprehension, ease of use, and time required to complete the questionnaires.

### Analysis

Categorical variables were described as frequencies and percentages and quantitative variables as the mean and standard deviation (SD). All analyses were performed using Microsoft Excel for Microsoft 365 MSO, version 2201, or R statistical package, version 4.0.3. The responses on barriers and facilitators were analyzed through textual analysis, classifying them according to the five core components of the model.

## Results

### Initial Screening of the Degree of Implementation of Integrated Social and Health Care HCS

A total of 105 (100.0%) SCS and 94 (25.0%) PHCs answered the screening questionnaire. The mean (SD) scores of the perception of the five core components of integrated social and health HCS were 1.4 (1.2) for SCS and 2.2 (1.3) for PHCs, which represented 27.5% and 40.7% of the maximum possible scores, respectively. The detailed scores for each core component are summarized in Table 2. Overall, PHC scores were higher than SCS scores for each of the core components. “Coordination between social and health multidisciplinary teams” was the component with the highest scores for both SCS and PHCs. In contrast, the components with the lowest scores were “individual assessment of integrated social and health care” and “single individual plan for integrated social and health care”, for SCS, and “shared protocols across health and social services” and “integrated portfolio of services with joint social and health HCS projects” for PHCs.

**Table 2:** Scores (0–5) of the screening questionnaire according to the five core components of integrated social and health HCS, answered by SCS and PHC social care professionals. *Mean (SD)*.


COMPONENTS	SCS (N = 105)	PHC (N = 94)

Individual assessment of integrated social and health care	1.2 (1.2)	2.3 (1.4)

Single individual plan for integrated social and health care	1.2 (1.1)	1.9 (1.4)

Shared protocols across health and social services	1.3 (1.1)	1.8 (1.3)

Coordination between social and health multidisciplinary teams	2.0 (1.2)	2.4 (1.4)

Integrated portfolio of services with joint social and health HCS projects	1.4 (1.3)	1.8 (1.3)


HCS, home care services; SCS, social care services; PHC primary health center.

Regarding the overall perception of the degree of implementation of integrated social and health HCS, SCS and PHC professionals rated it with mean (SD) scores of 2.7 (2.3) and 4.5 (2.6), respectively, thus representing 27.0% and 44.5% of the maximum possible scores, which are similar results to those found for the mean (SD) scores of the five core components.

### Identification of Indicators and Measurement Elements of the Core Components of Integrated Social and Health HCS

**Figure 2:**
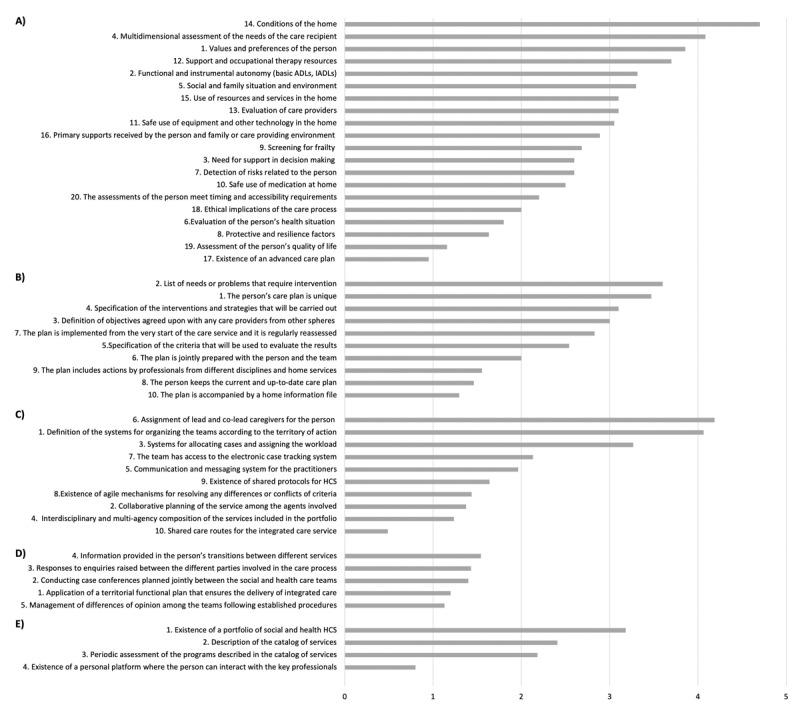
Mean scores of the indicators for each of the five core components of an integrated care model: **A)** “individual assessment of integrated social and health care” (n = 21); **B)** “single individual plan for integrated social and health care” (n = 18); **C)** “shared protocols across health and social services” (n = 17); **D)** “coordination between social and health multidisciplinary teams” (n = 19); **E)** “integrated portfolio of services with joint social and health HCS projects” (n = 11). Indicators are ordered from highest to lowest mean scores obtained from the individualized questionnaires answered by social care professionals.

A total of 48 (45.7%) SCS showed scores ≥3 in at least one core component of the screening questionnaire and, therefore, received a customized questionnaire to assess the degree of compliance of the indicators and measurement elements for those core components. Specifically, the number of SCS that rated the core components ≥3 were: 21 (20.0%), for the core component “individual assessment of integrated social and health care”; 18 (17.1%), for “single individual plan for integrated social and health care”; 17 (16.2%), for “shared protocols across health and social services”; 19 (18.1%), for “coordination between social and health multidisciplinary teams”; and 11 (10.5 %), for “integrated portfolio of services with joint social and health HCS projects”. These SCS gave a mean (SD) score of 3.0 (1.8), 2.7 (1.6), 2.2 (1.9), 1.4 (1.7), and 2.1 (1.9) for the component “individual assessment of integrated social and health care”, “single individual plan for integrated social and health care”, “shared protocols across health and social services”, “coordination between social and health multidisciplinary teams”, and “integrated portfolio of services with joint social and health HCS projects”, respectively. The mean scores for the indicators of each core component are shown in Figure 2.

The highest rated indicators for each core component were: “conditions of the home”, for the component “individual assessment of integrated social and health care” (Figure 2A); “list of needs or problems that require intervention”, for the component “single individual plan for integrated social and health care” (Figure 2B); “assignment of lead and co-lead caregivers for the person”, for the component “shared protocols across health and social services” (Figure 2C); “information provided in the person’s transitions between different services”, for the component “coordination between social and health multidisciplinary teams” (Figure 2D); and “existence of a portfolio of social and health HCS”, for the component “integrated portfolio of services with joint social and health HCS projects” (Figure 2E). Conversely, the lowest-scored indicators for each of the components above were: “existence of an advanced care plan”, for the component “individual assessment of integrated social and health care” (Figure 2A); “the plan is accompanied by a home information file specifying the key agreements and aspects to be taken into account in relation to the care recipient and their family”, for the component “single individual plan for integrated social and health care” (Figure 2B); “shared care routes for the integrated care service”, for the component “shared protocols across health and social services” (Figure 2C); “management of differences of opinion among the teams in accordance with established procedures”, for the component “coordination between social and health multidisciplinary teams” (Figure 2D); and “existence of a personal platform or folder where the person and caregiver can interact with the key professionals”, for the component “integrated portfolio of services with joint social and health HCS projects” (Figure 2E). The detailed results including all measurement elements are shown in Tables S1–S5 (Supplementary file 1). No differences in geographic areas or size of the counties were found for these 48 SCS showing scores ≥3 in at least one core component of the screening questionnaire.

### Identification of the Main Barriers and Facilitators of Integrated Social and Health HCS

The identified barriers and facilitators of integrated social and health HCS are summarized in Table 3. The lack of 1) shared protocols and culture of coordination, 2) staff and leadership, 3) shared information procedures between social and health care systems, and 4) agreement between social and health care professionals were the most frequent barriers for integrated social and health HCS. In contrast, recognizing the importance and need of integrated HCS, having previous collaboration experiences between social and health care areas, and a small territory of action were the most frequently reported facilitators.

**Table 3:** Main perceived barriers and facilitators of integrated HCS according to the responses of the screening and customized questionnaires by SCS (n = 105) and PHC (n = 94) social care professionals, *N (%)*.


BARRIERS	

Lack of shared protocols and culture of coordination between social and health care systems	93 (46.7)

Lack of staff, leadership, and responsibility	91 (45.7)

Lack of shared information procedures between social and health care systems	60 (30.1)

Lack of agreement and differences between social and health care professionals; work pressure	50 (25.1)

Fragmentation and different territorial organization between social and health care systems	23 (11.6)

Services portfolio not shared between social and health care systems; HCS limitations	22 (11.1)

Barriers for user assistance: duplicity, bureaucracy, complexity, and COVID-19 pandemic	18 (9.0)

Lack of resources and services	16 (8.0)

Data Protection issues that make difficult data collection and sharing	12 (6.0)

Lack of training regarding the integrated care program; lack of importance of social assessment in the health care field	9 (4.5)

**FACILITATORS**	

Good attitude, competence, and acknowledgment of the need for an integrated care approach by social and health care professionals	162 (81.4)

Previous experience in collaboration and common methods for networking	81 (40.7)

Small size of the territory of action and the community network	33 (16.6)

Cooperation of the users and their family members; social and health systems work with the same persons	12 (6.0)

Good attitude towards collaboration between social and health care systems	12 (6.0)

Specific training on the integrated HCS program	10 (5.0)

Technology tools that promote data sharing	9 (4.5)

Increased funds and creation of spaces for HCS	8 (4.0)

Regulation for the integration of social and health data	4 (2.0)

Unification of HCS with shared funds between social and health care systems	1 (0.5)


COVID-19, coronavirus disease 2019; HCS, home care services. SCS, social care services; PHC, primary health center.

## Discussion

This is the first study on the degree of integration of social and health HCS in Catalonia based on the social care workers’ perception. The questionnaires used in this analysis helped identify the perceived best and worst implemented components of social and health HCS integration, as well as its main barriers and facilitators.

We found that 100% of SCS and 25% of PHCs answered the screening questionnaire on the perception of the degree of implementation of integrated social and health HCS. This considerable difference in the response rates may be due to the burden of the COVID-19 pandemic for the health care system in 2020 and 2021, which has probably conditioned the chances of answering the questionnaire by PHC professionals. Nevertheless, a total of 96 (25%) PHCs representing different Catalan counties completed the questionnaire, even though participation in the study was voluntary, which suggests that they were interested in the integration of social and health HCS. The excellent response rate by SCS professionals (100%) probably relies on the thorough follow-up of the assessment team and the availability of the e-mail addresses of all their participants, which allowed them to send individualized reminders. Conversely, in PHCs, the reminder e-mail was sent to the social work reference professionals who were responsible for forwarding it to each participant. Besides, the SCS response rate can also be attributed to a great interest in the integration of social and health HCS. In this regard, most of the work of social services is related to HCS, whereas, in primary care, HCS only represent a small workload.

The scores of the screening questionnaire given by PHCs were higher than those given by SCS. Although the scores of SCS and PHCs are not statistically comparable due to the disparity in the response rates, these differences may be explained by the professional profile of the persons who answered the questionnaire in PHCs –PHC social workers, who have greater access to the information shared by social and health care systems– instead of the PHC team as a whole and a potential bias induced by a higher motivation of those PHC social workers that answered the questionnaire, resulting in higher scores. Nevertheless, either SCS or PHC scores showed clear opportunities for improvement in the integration of social and health HCS. In this regard, previous studies analyzing the degree of implementation of integrated social and health programs in European countries also observed a lack of integrated care implementation [27,28]. However, those programs were addressed to different users and settings and used different measures, precluding their direct comparison with this study’s results.

With respect to the scores obtained for each of the five core components of integrated care, both SCS and PHC teams agreed on assigning the highest integration scores to “coordination between social and health multidisciplinary teams”. Conversely, the lowest-rated components by SCS and PHCs were different: “individual assessment of integrated social and health care” and “single individual plan for integrated social and health care” for SCS, and “shared protocols across health and social services” and “integrated portfolio of services with joint social and health HCS projects” for PHCs. However, the lack of standardized tools, common language, and shared information procedures between social and health care teams make interpreting these differences difficult. In relation to the lowest-rated components by SCS, having a joint assessment and an integrated care plan (one person, one plan) is critical, but translating this plan to a unique Information and Communication Technology platform where both health and social care services could work is a challenging task.

Regarding the perceived barriers and facilitators of integrated care between social and health care services, the lack of 1) shared protocols and culture of coordination, 2) staff and leadership, 3) shared information procedures between social and health care systems, and 4) agreement between social and health care professionals were the barriers most frequently identified, whereas recognizing the importance and need of integrated HCS, having previous collaboration experiences between social and health care areas, and a small territory of action were the most common facilitators. Other studies reported similar perceived facilitators and barriers as those found in ours. For instance, Lewis et al. recently reported poor professional engagement and data sharing problems as frequent barriers to integrated care implementation, whereas effective senior leadership, shared values and vision, and a good level of funding were identified as common facilitators [29]. Cameron et al. found that difficulty in recruiting staff; lack of strong and appropriate management; differences in professionals’ philosophies, values, and cultures; difficulty in information sharing; and financial uncertainty were factors hindering integrated care. They also reported that understanding the aims of joint work and the roles and responsibilities of the professionals involved, previous joint working, and effective mechanisms to share information were facilitators of integrated care [30]. Urizar et al. described that non-integrated information systems, an individualist professional culture, working in silos, lack of training, and lack of systemic and integrative vision were barriers to implementing integrated care pathways. Conversely, better information systems, data collection improvement, and training health care professionals on communication and availability of professionals and teamwork skills were identified as facilitators of integrated care implementation [31]. All these barriers and facilitators are consistent with those found in our study. However, despite Auschra describing some of the inter-organizational barriers to collaboration in healthcare settings [32], we believe there is a lack of studies describing the barriers and facilitators of integrated services specifically in the home environment. Genet et al. mentioned some of them, but it was an initial, narrative study [9].

Our work has some limitations. First, the low response rate and representativeness in answering the screening questionnaire by PHC professionals limited the gathering of data on the implementation of integrated social and health HCS in PHCs. Besides, the second questionnaire required more time to be answered than the first one; hence, it was not sent to the PHCs because they were considerably burdened with the COVID-19 pandemic. Instead, SCS teams were asked to answer it together with PHC teams of their council to the extent possible. Therefore, our results mainly reflect the SCS social workers’ perception of the degree of implementation of social and health HCS, which does not include all social care teams responsible for social and health HCS in Catalonia. In addition, the low representativeness may have introduced a bias in the PHC scores of the screening questionnaire, resulting from a higher motivation of those PHC professionals who answered it. Another limitation may arise from the choice of the core components of an integrated model, as there are no previous studies that validate or support this choice. However, given the comprehensive review of previous documents on integrated care services and the stakeholders’ high participation in the model’s development, we may assume that our results could have face validity. In this regard, there is not a single method to evaluate integrated care between social and health care services, and a large number of measures have been reported across different studies [33]. Thus, the lack of standardization in the evaluation of integrated care services makes it difficult to contextualize our results. Moreover, in our study, the assessments were not validated externally. Therefore, in PHCs they were limited to the perception of social workers and lacked that of other health care professionals that also participate in HCS and must coordinate with SCS (e.g., nurses or general practitioners). However, despite these limitations, to our knowledge, this is the first study specifically assessing the degree of implementation along with the barriers and facilitators of integrated social and health HCS. Although our results may not apply to other systems supplying integrated social and health care, they provide some valuable insights for other countries regarding the implementation of integrated care in the home setting, especially in terms of the components that need to be strengthened in order to improve the integrated care model.

In conclusion, our study shows that, according to the selected five core components of an integrated model (individual assessment of integrated social and health care, single individual plan for integrated social and health care, shared protocols across health and social services, coordination between social and health multidisciplinary teams, and integrated portfolio of services with joint social and health HCS projects), the perceived degree of implementation of integrated health and social HCS offered by the Government of Catalonia is low and has a clear opportunity for improvement. In addition, the main barriers and facilitators of HCS are similar to those reported in other analyses. Future studies should include the assessment of different professionals of PHCs involved in HCS besides social workers, external audits, and further information on counties with low-level screening responses in order to increase the accuracy of the results. Despite the limitations of the study, our results have allowed us to launch a project to assess the impact of integrated social and health HCS in the areas with high integration scores compared with those with low integration scores, which we expect to publish in the near future.

## Data Accessibility Statement

The datasets used and/or analyzed during the current study are available from the corresponding author upon reasonable request.

## Additional Files

The additional files for this article can be found as follows:

10.5334/ijic.7530.s1 Supplementary File 1. Tables S1 to S5.

10.5334/ijic.7530.s2 Appendices. Appendix 1 and 2.
