# Supplementary material for: Integrated Health and Social Home Care Services in Catalonia: Professionals’ Perception of its Implementation, Barriers, and Facilitators
Source: Int J Integr Care. 2024 Apr 26;24(2):10. doi: 10.5334/ijic.7530 (PMC11049598; doi:10.5334/ijic.7530)
Supplement: Supplementary File 1. — Tables S1 to S5. [file ijic-24-2-7530-s1.pdf]

# Supplementary file 1

**Table S1.** Degree of compliance scores of measurement components of the element "individual assessment of integrated social and health care" in SCS according to customized questionnaire scores[a]. *Mean (SD).* N=21[b].

| | |
|---|---|
| **1. Values and preferences of the person** | 3.9 (1.0) |
| **2. Functional and instrumental autonomy (basic ADLs, IADLs) (n=19)** | 3.3 (0.7) |
| **3. Need for support in decision making (n=20)** | 2.6 (1.2) |
| **4. Multidimensional assessment of the needs of the care recipient (n=12)** | 4.1 (1.2) |
| **5. Social and family situation and environment** | 3.3 (1.2) |
| Basic social assessment (n=20) | 3.7 (0.9) |
| Social diagnoses | 3.2 (1.3) |
| Evaluation of social complexity criteria (n=20) | 3.7 (0.9) |
| The person's support network | 3.1 (1.3) |
| Dynamics of family relationships | 3.2 (1.3) |
| Interaction with the community | 2.8 (1.4) |
| Socioeconomic status and resources of the person and family in situations in which services not covered by universal health insurance are expected | 3.2 (1.3) |
| **6. Evaluation of the person's health situation (n=18)** | 1.8 (1.9) |
| System-based assessment (n=15) | 1.9 (1.2) |
| Health diagnoses (n=18) | 1.8 (2.2) |
| Identification and/or signs of frailty (n=18) | 2.3 (1.7) |
| Categorization of complex patients (CCPs/ACPs) (n=18) | 2.0 (2.2) |
| Specific assessment tailored to each group (n=17) | 1.7 (2.2) |
| Assessment of the presence of pain (n=16) | 1.3 (1.8) |
| Assessment of other symptoms (e.g., dyspnea) (n=16) | 1.4 (2.0) |
| **7. Detection of risks related to the person** | 2.6 (1.6) |
| Falls | 2.5 (1.6) |
| Mistreatment, neglect, or abuse | 3.4 (1.3) |
| Pressure ulcers (n=19) | 2.4 (1.6) |
| Unwanted loneliness | 3.0 (1.2) |
| Self-harm (n=20) | 1.8 (2.0) |
| Other (n=8) | 4.0 (1.1) |
| **8. Protective and resilience factors (n=19)** | 1.6 (1.8) |
| **9. Screening for frailty (n=19)** | 2.7 (1.3) |
| **10. Safe use of medication at home** | 2.5 (1.9) |
| Review and reconciliation of medication in the home | 2.8 (1.8) |
| Adherence to treatment (n=19) | 2.4 (1.7) |
| Systems for preparing and preserving the medication | 2.4 (2.3) |
| **11. Safe use of equipment and other technology in the home (n=19)** | 3.1 (1.3) |
| **12. Support and occupational therapy resources (n=10)** | 3.7 (1.3) |
| Screening for support product needs (n=10) | 4.0 (1.1) |
| Availability of occupational therapy (n=10) | 3.0 (1.9) |
| Occupational therapy assessment (n=10) | 3.0 (1.9) |
| Bank of technical products in the territory (n=10) | 4.2 (0.8) |
| **13. Evaluation of care providers** | 3.1 (1.4) |

| | |
|---|---|
| Key contact persons and caregivers | 3.4 (1.4) |
| Assessment of needs (n=20) | 2.7 (1.7) |
| Risk of claudication | 3.1 (1.3) |
| **14. Conditions of the home** | 4.7 (0.6) |
| Habitability | 4.6 (0.7) |
| Accessibility | 4.6 (0.7) |
| State of cleanliness | 4.8 (0.4) |
| Needs for adaptation of the environment | 4.4 (1.2) |
| Other (n=11) | 5.0 (0.0) |
| **15. Use of resources and services in the home: remote assistance, home health workers, respiratory physiotherapy, physical/occupational therapy, home oxygen therapy, speech therapy, etc. (n=20)** | 3.1 (1.2) |
| **16. Primary support received by the person and family or care providing environment (n=18)** | 2.9 (1.2) |
| **17. Existence of an advanced care plan, especially in the case of ACPs (n=20)** | 1.0 (1.6) |
| **18. Ethical implications of the care process (n=19)** | 2.0 (1.4) |
| **19. Assessment of the person's quality of life, conducted using a quality-of-life assessment tool or scale (n=19)** | 1.2 (1.7) |
| **20. The initial and subsequent assessments of the person meet timing and accessibility requirements (n=19)** | 2.2 (1.6) |
| The assessment of the person is unique and supplemented by health and social care professionals (n=18) | 1.6 (2.0) |
| The record of the assessment is available to both social and health care professionals at the person's own home in order for them to view it (n=19) | 0.6 (1.5) |
| The assessment is conducted during the first two weeks after joining the Integrated Social and Health HCS Program (n=18) | 0.9 (1.5) |
| The assessment includes a diagnostic face-to-face visit to the person's home (n=19) | 3.9 (1.4) |
| Periodic assessments are carried out at least annually or whenever the person's situation changes significantly (n=19) | 3.9 (1.5) |

[a]Scores range: 0 to 5. [b]Unless otherwise specified, the assessment corresponds to the 21 SCS that rated the attribute "individual assessment of integrated social and health care" with a score ≥3 in the screening questionnaire.

ACPs, advanced chronic patients; ADLs, activities of daily living; CCPs, complex chronic patients; HCS, home care services; IADLs, instrumental activities of daily living; SCS, social care services.

**Table S2.** Degree of compliance scores of measurement components of the element "single individual plan for integrated social and health care" in SCS according to customized questionnaire scores[a]. *Mean (SD).* N=18[b].

| | |
|---|---|
| **1. The person's care plan is unique** | 3.5 (0.9) |
| **2. List of needs or problems that require intervention (n=17)** | 3.6 (1.0) |
| Specification of the priority problems of the person and their family and main caregiver (n=17) | 3.5 (0.8) |
| **3. Definition of objectives agreed upon with any care providers from other spheres** | 3.0 (1.1) |
| Expectations and objectives of the person and their family regarding the care process | 2.6 (1.1) |
| **4. Specification of the interventions and strategies that will be carried out (n=17)** | 3.1 (1.2) |
| Specification of the professionals or disciplines responsible for their execution (n=17) | 3.0 (1.2) |
| **5. Specification of the criteria that will be used to evaluate the results** | 2.5 (1.0) |
| **6. The plan is jointly prepared with the person and the team** | 2.0 (1.8) |
| It is based on the interdisciplinary and integrated assessment of the person and their environment | 2.0 (2.1) |
| It is prepared together with the person, the social and health interdisciplinary team and the person's key support network when necessary | 1.8 (2.0) |
| It is prepared through a process of shared decision-making | 2.3 (1.4) |
| **7. The plan is implemented from the very start of the care service, and it is reassessed within the first 6 weeks and at least once a year (n=16)** | 2.8 (1.2) |
| **8. The person can view the plan and keeps the current and up-to-date care plan (n=17)** | 1.5 (1.3) |
| **9. The plan includes actions by the professionals from the various disciplines and home services that visit the person at home (n=17)** | 1.6 (2.0) |
| **10. The plan is accompanied by a home information file specifying the key agreements and aspects to be taken into account in relation to the care recipient and their family** | 1.3 (1.9) |

[a]Scores range: 0 to 5. [b]Unless otherwise specified, the assessment corresponds to the 18 SCS that rated the attribute "single individual plan for integrated social and health care" with a score ≥3 in the screening questionnaire.
SCS, social care services.

**Table S3.** Degree of compliance scores of measurement components of the element "shared protocols across health and social services" in SCS according to customized questionnaire scores[a]. *Mean (SD).* N=17[b].

| | |
|---|---|
| **1. Definition of the systems for organizing the teams according to the territory of action (n=16)** | 4.1 (1.6) |
| **2. Collaborative planning of the service among the agents involved (n=16)** | 1.4 (1.7) |
| **3. Systems for allocating cases and assigning the workload (n=16)** | 3.3 (1.6) |
| **4. Interdisciplinary and multi-agency composition of the services included in the portfolio of the home care teams** | 1.2 (1.9) |
| **5. Communication and messaging system for the practitioners involved in the care process (n=16)** | 2.0 (1.4) |
| **6. Assignment of lead and co-lead caregivers for the person (n=16)** | 4.2 (1.4) |
| **7. The team has access to the electronic case tracking system (n=14)** | 2.1 (1.3) |
| **8. Existence of agile mechanisms for resolving any differences or conflicts of criteria arising between professionals and organizations** | 1.4 (1.8) |
| **9. Existence of shared protocols for HCS (n=5)** | 1.6 (1.4) |
| **10. Shared care routes for the integrated care service (n=15)** | 0.5 (1.0) |

[a]Scores range: 0 to 5. [b]Unless otherwise specified, the assessment corresponds to the 17 SCS that rated the attribute "shared protocols across health and social services" with a score ≥3 in the screening questionnaire.
HCS, home care services; SCS, social care services.

**Table S4.** Degree of compliance scores of measurement components of the element "coordination between social and health multidisciplinary teams" in SCS according to customized questionnaire scores[a]. *Mean (SD).*N=19[b].

| | |
|---|---|
| **1. Existence and application of a territorial functional plan that ensures the delivery of integrated care (n=8)** | 1.2 (1.7) |
| Application of the plan regarding the continuity of care with primary health care (n=6) | 0.8 (1.4) |
| Application of the plan for continuity of care with the social-health care network (n=7) | 1.2 (1.8) |
| Application of the plan for continuity of care with key hospitals (n=7) | 1.2 (1.8) |
| Application of the plan for continuity of care with other specialized services (n=7) | 1.1 (1.5) |
| Incorporation of volunteers and other community initiatives (n=8) | 1.5 (1.6) |
| **2. Conducting case conferences planned jointly between the social and health care teams** | 1.4 (1.8) |
| **3. Responses to enquiries raised between the different parties involved in the care process** | 1.4 (2.0) |
| **4. Information provided in the person's transitions between different services** | 1.5 (1.9) |
| **5. Management of differences of opinion among the teams in accordance with established procedures (n=18)** | 1.1 (1.6) |

[a]Scores range: 0 to 5. [b]Unless otherwise specified, the assessment corresponds to the 19 SCS that rated the attribute "coordination between social and health multidisciplinary teams" with a score ≥3 in the screening questionnaire.
SCS, social care services.

**Table S5.** Degree of compliance scores of measurement components of the element “integrated portfolio of services with joint social and health HCS projects” in SCS according to customized questionnaire scores[a]. *Mean (SD).* N=11[b].

| | |
|---|---|
| **1. Existence of a portfolio of social and health HCS** | 3.2 (1.9) |
| **2. Description of the catalog of services** | 2.4 (1.9) |
| **3. Periodic assessment of the programs described in the catalog of services** | 2.2 (1.6) |
| **4. Existence of a personal platform or folder where the person and caregiver can interact with the key professionals (n=10)** | 0.8 (1.3) |

[a]Scores range: 0 to 5. [b]Unless otherwise specified, the assessment corresponds to the 11 SCS that rated the attribute “integrated portfolio of services with joint social and health HCS projects” with a score ≥3 in the screening questionnaire.
HCS, home care services; SCS, social care services.
