# Supplementary material for: Integrated Health and Social Home Care Services in Catalonia: Professionals’ Perception of its Implementation, Barriers, and Facilitators
Source: Int J Integr Care. 2024 Apr 26;24(2):10. doi: 10.5334/ijic.7530 (PMC11049598; doi:10.5334/ijic.7530)
Supplement: Appendices. — Appendix 1 and 2. [file ijic-24-2-7530-s2.pdf]

Appendices

Appendix 1. INITIAL SCREENING QUESTIONNAIRE

Invitation to participate in the baseline assessment of the state of integrated home care services in Catalonia

Dear ...,

Here at the General Directorate of Personal Autonomy and Disability of Catalonia, we wish to carry out a baseline or diagnostic assessment of the integrated social and health care services provided in the home environment in Catalonia. Through this assessment, we will identify people's experiences of integrated care services currently being provided, as well as the extent of their deployment and their impact, in order to identify areas for improvement. This assessment is being conducted in relation to the shared care services provided under the Integrated Social and Health Care Plan (CSHCP), overseen by the Strategic Primary Care Directorate of the Department of Health of Catalonia.

We believe this is an excellent opportunity to build a picture of the health and social needs of the population who receives home care services in your territory (including the Home Care Service, remote assistance, technical assistance, the Home Care Program, and other related services). The goal of this assessment project is not only to identify the degree to which the model is being implemented and to identify associated best practices, but also to conduct an analysis of the service's impact and identify key elements that will allow us to extend these practices to the rest of the system in the future.

In order to learn more about the integrated home care service initiatives in your territory, we would like to ask for your opinion in order to identify the degree of deployment of these integrated home care services from your perspective, as well as to identify any barriers, facilitators and best practices developed in your territory. The questionnaire will take you approximately 10 to 15 minutes to complete.

You can open the questionnaire and provide your assessment at the following link:

https://ec.europa.eu/eusurvey/runner/AVALUACIO_BASAL_PAID

The deadline for receiving your contributions is 15 February 2021.

If you have any questions, comments, or suggestions, please contact menfedaque.bcn.ics@gencat.cat or philarion@fadq.org.

Your participation will help us to obtain a picture – covering the whole of Catalonia – of your perception and opinion regarding these integrated home care services in your territory.

We would like to take this opportunity to express in advance our gratitude for your collaboration.

Aina Plaza Tesías

General Director of Personal Autonomy and Disability
General Directorate of Personal Autonomy and Disability of Catalonia
autonomiaidiscapacitat.tsf@gencat.cat

Rafael Ruiz Riera

Strategic Director
Departmental Commission for Strategic Drive in Primary Care and Community Health
Department of Health of Catalonia

Introduction to the questionnaire:

Thank you for accepting our invitation to participate in the baseline assessment of the Integrated Home Care Program.

The questionnaire contains 5 sections. It will take you approximately 10 to 15 minutes to complete.

At the end of each question, you will find a space for remarks in case you wish to add any comments. In order to facilitate the processing of the answers and their interpretation, we kindly ask you to be as clear and concise as possible in your answers, using short sentences and clear and specific language insofar as possible.

Your participation will help us to obtain a picture covering all of Catalonia of your perception and opinion regarding these integrated home care services in your territory.

Thank you for participating!

I. Identification details:

- Name of Social Care Services:
- Position:
- Service/Team:

II. Perception and opinion: rate from 0 to 5 the degree of implementation in your territory of the following key aspects of the integrated social and health home care services.

Note: "0" is no or minimal implementation, and "5" is the maximum desirable implementation in your territory

Core elements of the integrated social and health home care services	Score from 0 to 5	Remarks
**1. Individual assessment of integrated social and health care.** A comprehensive and integrated assessment is conducted using tools that are both unique to and shared between the two spheres when integrated care is required.		
**2. Single individual care plan.** An individual care plan is developed in a collaborative manner, suitable to the teams of professionals in both spheres.		
**3. Shared protocols across health and social services.** Collaborative work dynamic with shared protocols across health and social services in different formats (joint complexity route, 24/7 care, management of risks in the home environment, etc.).		
**4. Coordination between social and health multidisciplinary teams.** Spaces are created and time is devoted to the work shared between the two spheres, both reactively in terms of the capacity to respond to crisis situations, and proactively by establishing key individuals in both spheres for the		

recipient of the care (lead and co-lead caregivers), as well as implementing digital solutions that facilitate a collaborative service (integrated registration systems, shared messaging service, etc.).		
**5. Integrated portfolio of services with joint social and health home care projects.** Elements of the portfolio of home care services are incorporated. These are available to and used by both spheres, thereby facilitating direct access to resources and services for both spheres when a need is identified so that they can be prescribed and implemented.		

III. What score from 0 to 10 would you give to the implementation of the integrated social and health home care services in your territory?

IV. What would you consider the main barriers and facilitators of the integrated home care services in your territory?

Barriers	Facilitators

V. We attach a Word document in which you can **set out the best practices you are employing in your territory related to the integrated home care services**. You can share all practices which you consider to be scalable on the basis of their value in helping to produce better results.

Appendix 2. QUESTIONNAIRE TO ASSESS IN DETAIL THE DEGREE OF DEPLOYMENT OF THE MEASURES

Self-evaluation of the care processes related to the integrated social and health home care services

Name of Social Care Services:
Team or territory name:
Date:

Below you will find 5 tables for identifying your perception and opinion regarding the degree of development of the integrated care services in the following areas:

1. **Overall integrated evaluation**
2. **Individual and integrated care plan**
3. **Integrated care tools (shared protocols, routes, messaging, shared records, etc.)**
4. **Continuity of the care and coordination between multidisciplinary teams**
5. **Portfolio of integrated services and evaluation of the catalog of services**

Rate from 0 to 5 the degree of implementation in your territory, where "**0**" indicates no or minimal implementation and "**5**" the maximum desirable implementation in your territory as a whole.
Note: Answer the questions for which you have information.

**1. Overall integrated evaluation**	**Score from 0 to 5**	**Comments**
1. Values and preferences of the person		
2. Functional and instrumental autonomy (basic ADLs, IADLs)		
3. Need for support in decision making		
4. Multidimensional assessment of the needs of the care recipient		
5. Social and family situation and environment:		
• Basic social assessment		
• Social diagnoses		
• Evaluation of social complexity criteria		
• The person's support network		
• Dynamics of family relationships		
• Interaction with the community		
• Socioeconomic status and resources of the person and family in situations in which services not covered by universal health insurance are expected		
6. Evaluation of the person's health situation		

• System-based assessment		
• Health diagnoses		
• Identification and/or signs of frailty		
• Categorization of complex patients (CCPs/ACPs)		
• Specific assessment tailored to each group		
• Assessment of the presence of pain		
• Assessment of other symptoms (e.g., dyspnea)		
7. Detection of risks related to the person		
• Falls		
• Mistreatment, neglect, or abuse		
• Pressure ulcers		
• Unwanted loneliness		
• Self-harm		
• Other risks		
8. Protective and resilience factors		
9. Screening for frailty		
10. Safe use of medication at home		
• Review and reconciliation of medication in the home		
• Adherence to treatment		
• Systems for preparing and preserving the medication		
11. Safe use of equipment and other technology in the home		
12. Support and occupational therapy resources		
• Screening for support product needs		
• Availability of occupational therapy		
• Occupational therapy assessment		
• Bank of technical products in the territory		
13. Evaluation of care providers		
• Key contact persons and caregivers		
• Assessment of needs		
• Risk of claudication		
14. Conditions of the home		
• Habitability		
• Accessibility		
• State of cleanliness		
• Needs for adaptation of the environment		
• Other		
15. Use of resources and services in the home: remote assistance, home health workers, respiratory physiotherapy, physical/occupational therapy, home oxygen therapy, speech therapy, etc.		
16. Primary supports received by the person and		

family or care providing environment		
17. Existence of an advanced care plan, especially in the case of ACPs		
18. Ethical implications of the care process		
19. Assessment of the person's quality of life, conducted using a quality-of-life assessment tool or scale		
20. The initial and subsequent assessments of the person meet timing and accessibility requirements		
• The assessment of the person is unique and supplemented by social and health care professionals		
• The record of the assessment is available to both social and health care professionals at the person's own home in order for them to view it		
• The assessment is conducted during the first two weeks of the person joining the Integrated Home Care Program		
• The assessment includes a diagnostic face-to-face visit to the person's home		
• Periodic assessments are carried out at least annually or whenever the person's situation changes significantly		

ACPs, advanced chronic patients; ADLs, activities of daily living; CCPs, complex chronic patients; IADLs, instrumental activities of daily living.

Remarks:

**2. Individual and integrated care plan**	**Score from 0 to 5**	**Comments**
1. The person’s care plan is unique		
The plan specifies the following components:		
2. List of identified needs or problems that require intervention.		
• Specification of which are **the priority** problems of the person and their family and main caregiver		
3. Definition of objectives agreed upon with any care providers from other spheres		
• Expectations and objectives of the person and their family regarding the care process		
4. Specification of the interventions and strategies that will be carried out.		
• Specification of the professionals or the disciplines responsible for their execution		
5. Specification of the criteria that will be used to evaluate the results achieved through the plan.		
6. The plan is jointly prepared with the person and the team		
• It is based on the interdisciplinary and integrated assessment of the person and their environment		
• It is prepared with the person, the social and health interdisciplinary team, and the person’s key support network when necessary		
• It is prepared through a process of shared decision-making		
7. The plan is implemented from the very start of the care service provision, and a re-assessment of the plan is conducted within the first 6 weeks and at least once a year, so that it is kept constantly up-to-date.		
8. The person can view their plan and keeps the current and up-to-date care plan.		
9. The integrated care plan includes actions by the professionals from the various disciplines and services that visit the person at home.		
10. The plan is accompanied by a home information file, specifying the key agreements and aspects to be taken into account in relation to the care recipient and their family.		

Remarks:

**3. Integrated care tools (shared protocols, routes, messaging, shared records, etc.)**	**Score from 0 to 5**	**Comments**
The facilitating tools for the integrated home care service measured through this indicator are as follows:		
1. Definition of the systems for organizing the teams according to the territory of action		
2. Collaborative planning of the service among the various agents involved (case conferences, inquiries between teams, etc.).		
3. Systems for allocating cases (caseload of the teams) and assignment of the workload according to the amount of support required.		
4. Interdisciplinary and multi-agency composition of the various services included in the portfolio of the home care teams.		
5. Existence of a communication and messaging system for the practitioners involved in the care process.		
6. Assignment of lead and co-lead caregivers for the recipient of the integrated care service.[1]		
7. The team has access to the Integrated Home Care Program’s electronic case tracking system, activated by the program’s professionals, with the possibility to incorporate multi-platform teleconferences, images, and transmissions.		
8. Existence of agile mechanisms for resolving any differences or conflicts of criteria arising between professionals and organizations.		
9. Existence of shared protocols for the home care service (to unify the minimum protocols with the Project Monitoring Commission)		
10. Shared care routes for the integrated care service (e.g., complexity route)		

Remarks:

[1] Deploying the integrated care service must offer the recipient a qualitative leap, based on home care provided by an interdisciplinary team which must include the workers familiar to the person as an integral part of that team. For this reason, it is necessary to define which individuals may be assigned as the lead and co-lead caregivers.

**4. Continuity of the care and coordination between multidisciplinary teams**	**Score from 0 to 5**	**Comments**
1. Existence and application of a territorial functional plan to ensure delivery and continued care under the integrated home care service		
• Application of the functional plan in relation to the continuity of care with primary health care		
• Application of the functional plan for continuity of care with the social-health care network		
• Application of the functional plan for continuity of care with key hospitals		
• Application of the functional plan for continuity of care with other specialized services, defining the process for coordination of the integrated home care service		
• Incorporation of volunteers and other community initiatives within the framework of the integrated home care service		
2. Conducting case conferences or discussions of cases planned jointly between the social and health care teams (either face-to-face or virtually)		
3. Responses to inquiries raised between the different parties involved in the care process (with response times within the established limits)		
4. Information provided in the person's transitions between different services		
5. Management of differences of opinion among the teams in accordance with established procedures.		

Remarks:

**5. Portfolio of integrated services and evaluation of the catalog of services**	**Score from 0 to 5**	**Comments**
1. A portfolio of integrated home care services exists		
2. A brief description of the catalog of services exists, including, as a minimum, a description of the service in question, the objectives, the profile of the users to whom the activities or services are offered, the operating hours, and the personnel responsible for the functional equipment. Activities are described at the individual, family, and community level.[2]		
3. As part of the Integrated Home Care Program in the territory, an assessment is conducted periodically (at least annually) of the programs described in the integrated catalog of services of all the parties involved in order to identify any need to adapt the program, as well as any other areas for improvement.		
4. Existence of a personal platform or folder where the user and caregiver can interact with the key professionals		

Remarks: [3]

[2] An alternative classification of the activities could be: preventive, assistance, educational, and community.

[3] **ADDITIONAL COMMENTS:** Below is an explanatory scale setting out the meanings of the scores from 0 to 5, which you may find useful when making your assessments.

- The score "0" indicates the minimum score, or not implemented.
- The score "1" indicates an initial phase. The processes are not usually documented, and operations are reactive or action is taken when a specific case arises.
- The score "2" indicates partially developed. Plans have been established: a strategy has been developed and its implementation has begun, although the processes are inconsistent. This assessment or measurement applies to 30% or more of the people tended to.
- The score "3" indicates an established process: defined, standard processes in force, used systematically, susceptible to improvement over time. This assessment applies to 60% or more of the people tended to.
- The score "4" indicates a good level of development. Mature: processes have been tested under variable conditions over a period of time, and their impact is beginning to show. This assessment applies to 90% or more of the people tended to.
- The score "5" indicates an excellent level of exemplary development: fully integrated into the system and the results for the recipients generally reflect this, with continuous improvement driven by incremental and innovative changes. It would be a best practice to be scaled and implemented throughout Catalonia.
